# Analysis of childhood overweight and obesity in Namibia using spatio-temporal quantile interval models

**DOI:** 10.1186/s41043-021-00274-7

**Published:** 2021-12-02

**Authors:** Owen Paweni Loss Mtambo, Legesse Kassa Debusho

**Affiliations:** 1 Department of Public Health, Kamuzu University of Health Sciences, Blantyre, Malawi; 2 Department of Statistics, University of South Africa, Pretoria, South Africa

**Keywords:** DIC, Fully Bayesian inference, Log-score, Obesity, Overnutrition, Overweight, Quantile interval regression, R-INLA, Spatio-temporal modell, Stunting, WAIC

## Abstract

The global prevalence of overweight (including obesity) in children under 5 years of age was 7% in 2012, and it is expected to rise to 11% by the year 2025. The main objective of this study was to fit spatio-temporal quantile interval regression models for childhood overweight (including obesity) in Namibia from 2000 to 2013 using fully Bayesian inference implemented in R-INLA package in R version 3.5.1. All the available Demographic and Health Survey (DHS) datasets for Namibia since 2000 were used in this study. Significant determinants of childhood overweight (including obesity) ranged from socio-demographic factors to child and maternal factors. Child age and preceding birth interval had significant nonlinear effects on childhood overweight (including obesity). Furthermore, we observed significant spatial and temporal effects on childhood overweight (including obesity) in Namibia between 2000 and 2013. To achieve the World Health Organisation (WHO) global nutrition target 2025 in Namibia, the existing scaling-up nutrition programme and childhood malnutrition policy makers in this country may consider interventions based on socio-demographic determinants, and spatio-temporal variations presented in this paper.

## Background

The common indicators of childhood overnutrition are overweight and obesity. Overweight and obese children have higher risk of suffering from diabetes, asthma, sleep disorder, high blood pressure, liver disease, and other diet-related noncommunicable diseases (NCD) [1].

The overall World Health Organisation (WHO) global nutrition target 2025 is to improve maternal, infant, and young child nutrition. One of the specific nutrition targets is the policy of overweight and obesity which aims at making sure that there is no more increase in prevalence of childhood overweight and obesity from 2014 to 2025 [1]. To attain this specific nutrition target, scaling-up nutrition programmes are available in most countries across sub-Saharan Africa (SSA) including Namibia [2]. The main aim of this research was to assess socio-demographic determinants and geographical variation of childhood overweight (including obesity) in Namibia between 2000 and 2013 using spatio-temporal quantile interval regression models implemented in R-INLA package [3] in R version 3.5.1.

To best of our knowledge, most studies on quantile modelling have emphasised on selecting only one specific response quantile level of interest and report the recommendations based on the only chosen response quantile. In our current study, we used quantile interval modelling approach which is more efficient because it uses weighted mean estimates based on quantile levels in a specified quantile interval of interest.

The rest of this paper is arranged as follows. The study population, sources of data, proposed quantile interval regression estimation method, and data analysis procedures are introduced in “Materials and methods” section. The results and their discussions are presented in “Results” and “Discussion” sections, respectively. Finally, conclusions and recommendations are given in “Conclusions” section.

## Materials and methods

### Study population

**Fig. 1:**
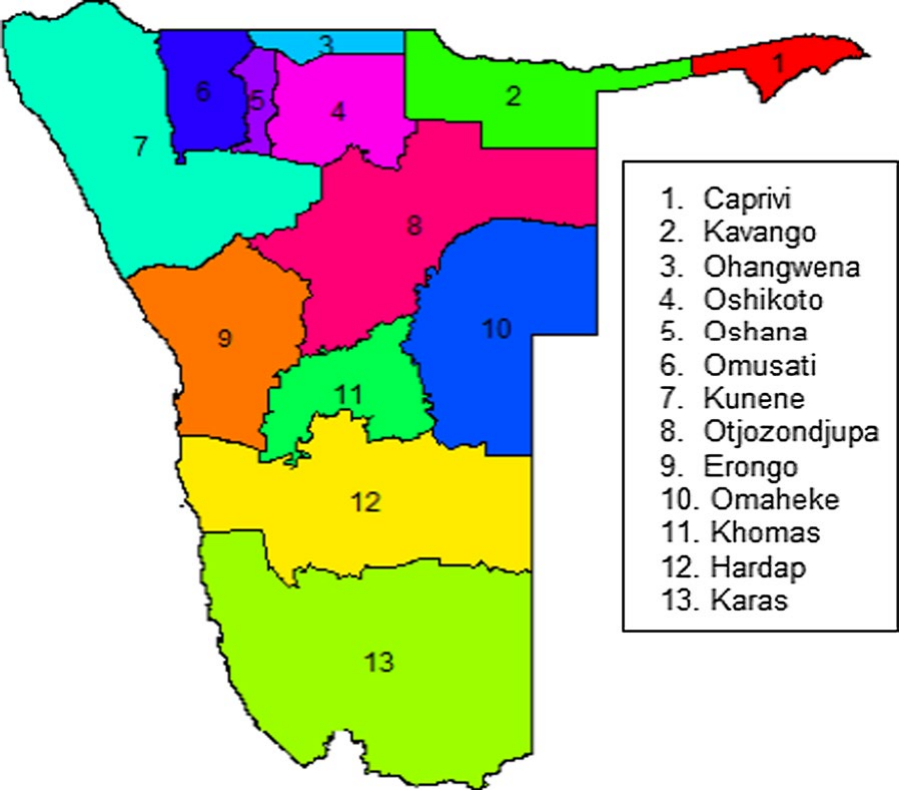
Map of Namibia with only 13 political regions by year 2013

The target population for this study was the entire country of Namibia located in the South-Western part of Africa. The country comprises of 14 political regions; Zambezi (formerly Caprivi), Erongo, Hardap, Karas, Kavango East, Kavango West, Khomas, Kunene, Ohangwena, Omaheke, Omusati, Oshana, Oshikoto, and Otjozondjupa. However, by 2013, Namibia had only 13 political regions until August 2013 when Kavango region was split into two regions; Kavango East and Kavango West. Figure 1 shows the map of Namibia with 13 political regions considered in this paper.

### Data sources

For applications of the newly proposed methodology, we considered all available Demographic and Health Survey (DHS) datasets for Namibia from 2000 to 2013. The primary outcome in this study was the childhood overweight (including obesity) in Namibia between 2000 and 2013 which was assessed by using the childhood body mass index-for-age $z$-score (BMIAZ).

### Quantile interval estimation

Suppose that the quantile interval of interest is given by ${\tau }^{*}\pm \Delta {\tau }^{*}=[{\tau }^{*}-\Delta {\tau }^{*}, {\tau }^{*}+\Delta {\tau }^{*}]$, where ${\tau }^{*}$ is the desired quantile interval median, and $\Delta {\tau }^{*}>0$ is the desired quantile bandwidth. The quantile interval weighted estimates for $(p+1)$ fixed effects parameters denoted by ${\widehat{\beta }}_{i}({\tau }^{*}\pm \Delta {\tau }^{*})$ for $i=0,1,2,\dots ,p$ and $q$ smoothing functions for nonlinear and spatio-temporal effects denoted by ${\widehat{g}}_{i}({\tau }^{*}\pm \Delta {\tau }^{*})$ for $i=1,2,\dots ,q$ are estimated as follows.

Firstly, compute the quantile interval weighted mean estimates for $(p+1)$ fixed effects parameters for each $i=0,1,2,\dots ,p$ using the formula

1 $$\hat{\user2{\beta }}_{{\varvec{i}}} \left( {{\varvec{\tau}}^{\user2{*}} \pm \Delta {\varvec{\tau}}^{\user2{*}} } \right) = \mathop \sum \limits_{{{\varvec{j}} = {\mathbf{0}}}}^{{\varvec{n}}} {\varvec{\omega}}_{{\varvec{j}}} \hat{\user2{\beta }}_{{{\varvec{ij}}}} \left( {{\varvec{\tau}}_{{\varvec{j}}}^{\user2{*}} } \right)$$

where ${{\varvec{\omega}}}_{{\varvec{j}}}=$ normalised ${\varvec{j}}$th weight assigned to ${{\varvec{\tau}}}_{{\varvec{j}}}^{\boldsymbol{*}}$ such that $\sum_{{\varvec{j}}={\mathbf{0}}}^{{\varvec{n}}}{{\varvec{\omega}}}_{{\varvec{j}}}={\mathbf{1}}$.

Secondly, compute the quantile interval weighted estimates for $q$ smoothing functions for each $i=1,2,\dots ,q$ using the formula

2 $${\widehat{{\varvec{g}}}}_{{\varvec{i}}}\left({{\varvec{\tau}}}^{\boldsymbol{*}}\pm \Delta {{\varvec{\tau}}}^{\boldsymbol{*}}\right)=\sum_{{\varvec{j}}={\mathbf{0}}}^{{\varvec{n}}}{{\varvec{\omega}}}_{{\varvec{j}}}{\widehat{{\varvec{g}}}}_{{\varvec{i}}{\varvec{j}}}({{\varvec{\tau}}}_{{\varvec{j}}}^{\boldsymbol{*}})$$

where ${{\varvec{\omega}}}_{{\varvec{j}}}=$ normalised ${\varvec{j}}$th weight assigned to ${{\varvec{\tau}}}_{{\varvec{j}}}^{\boldsymbol{*}}$ such that $\sum_{{\varvec{j}}={\mathbf{0}}}^{{\varvec{n}}}{{\varvec{\omega}}}_{{\varvec{j}}}={\mathbf{1}}$.

Using the 2006 World Health Organisation (WHO) growth standards for assessing childhood nutritional status, the cut-off points for childhood overweight and obesity are $2\le {\mathrm{BMIAZ}}\le 3$ and ${\mathrm{BMIAZ}}>3$, respectively [14]. In our current study, these cut-off points corresponded to quantile interval $[0.85, 0.99]$ for modelling overweight (including obesity). Consequently, our primary outcome was BMIAZ in the quantile interval ${\tau }^{*}\pm \Delta {\tau }^{*}=0.92\pm 0.07$ i.e. $[{\tau }^{*}-\Delta {\tau }^{*}, {\tau }^{*}+\Delta {\tau }^{*}]=[0.85, 0.99]$ where ${\tau }^{*}=0.92$ was the desired median, and $\Delta {\tau }^{*}=0.07$ was the desired bandwidth.

### Latent models and prior distributions

Since we used fully Bayesian framework, it was necessary to supplement all unknown functions $\left\{ g \right\}^{\prime}s$ for both nonlinear and spatio-temporal covariates, all parameters $\left\{ \beta \right\}^{\prime}s$ for categorical covariates and all variance parameters $\{ \sigma^{2} \} ^{\prime}s$ by appropriate latent models and prior distributions. Intuitively, the priors for all unknown functions usually belong to the class of Gaussian Markov random fields (GMRF). In this paper, we considered several GMRFs including first order random walk (RW1) modell, second order random walk (RW2) modell, intrinsic conditional autoregressive (ICAR) models, proper conditional autoregressive (PCAR) models, and identically and independently distributed (IID) models [4–6].

For the fixed effect parameters $\left\{ {\beta_{j} } \right\}^{\prime}s$, we supplemented either the Gaussian priors, or log-Gamma priors, or logit-Beta priors [7–9].

### Posterior inference

The intuitive method for estimating Bayesian posterior marginal distribution is Markov chain Monte Carlo (MCMC). The alternative method is integrated nested Laplace approximation (INLA) [3]. We used INLA method because it is generally faster and that the solution converges quickly than MCMC for quantile models [3, 10].

### INLA approach

The R-INLA [3] package of R software [11] was used for all data analyses. This package implements the INLA method, which performs the direct computation of the marginal posterior densities in a large latent Gaussian modell (LGM) sub-class of the fully Bayesian hierarchical models, instead of using the time-consuming MCMC simulation technique. The package is appropriate for all hierarchical models that have the following form.

$$\begin{aligned}&{\mathrm{Level }}\,1{:}\,\left({y}_{i}|{\varvec{x}},\boldsymbol{\vartheta }\right)\sim \pi \left({y}_{i}|{\eta }_{i},\boldsymbol{\vartheta }\right)\\&{\mathrm{Level }}\,2{:}\,\left({\varvec{x}}|\boldsymbol{\vartheta }\right)\sim N\left({\mathbf{0}},{{\varvec{Q}}}^{-1}(\boldsymbol{\vartheta })\right)\\&{\mathrm{ Level }}\,3{:}\,\boldsymbol{\vartheta }\sim \pi \left(\boldsymbol{\vartheta }\right)\end{aligned}$$

where $\boldsymbol{\vartheta }$ is a set of hyperparameters, ${\varvec{x}}$ is a latent Gaussian field, ${\varvec{y}}={\left({y}_{1},{y}_{2},\dots ,{y}_{n}\right)}^{\mathrm{T}}$ is a vector of responses, ${\eta }_{i}$ is a linear predictor for each individual $i$, and ${\varvec{Q}}(\boldsymbol{\vartheta })$ is the precision matrix to the latent filed ${\varvec{x}}$ conditioned on $\boldsymbol{\vartheta }$.

Basically, INLA method is used to approximate a desired marginal posterior density of $\boldsymbol{\vartheta }$ using a Gaussian approximation $\tilde{\pi }\left({\varvec{x}}|\boldsymbol{\vartheta },{\varvec{y}}\right)$ for the posterior on the latent field evaluated at the posterior mode, ${{\varvec{x}}}^{\boldsymbol{*}}\left(\boldsymbol{\vartheta }\right)=\mathop {\mathrm{argmax}}\nolimits_{{\varvec{x}}}\pi \left({\varvec{x}}|\boldsymbol{\vartheta },{\varvec{y}}\right),$ which is given by

3 $$\pi \left(\boldsymbol{\vartheta }|{\varvec{y}}\right)\propto {\left.\frac{\pi \left({\varvec{x}}\boldsymbol{\vartheta },{\varvec{y}}\right)}{\pi \left({\varvec{x}}|\boldsymbol{\vartheta },{\varvec{y}}\right)}\right|}_{x={{\varvec{x}}}^{\boldsymbol{*}}\left(\boldsymbol{\vartheta }\right)}\approx {\left.\frac{\pi \left({\varvec{x}},\boldsymbol{\vartheta },{\varvec{y}}\right)}{ \tilde{\pi }\left({\varvec{x}}|\boldsymbol{\vartheta },{\varvec{y}}\right)}\right|}_{{{\varvec{x}}={\varvec{x}}}^{\boldsymbol{*}}\left(\boldsymbol{\vartheta }\right)}$$

and is called the Laplace approximation [12–15].

The algorithm uses numerical optimisation to find mode of the posterior. The marginal posteriors of each ${x}_{j}$ and ${\vartheta }_{k}$ are then calculated using numerical integration over $\boldsymbol{\vartheta }$, with another Laplace approximation (hence nested Laplace) involved in the two latent field marginal posterior computations: $\pi \left({x}_{j} |{\varvec{y}}\right)\approx \int \tilde{\pi }\left({\varvec{x}}|\boldsymbol{\vartheta },{\varvec{y}}\right)\tilde{\pi }\left(\boldsymbol{\vartheta }|{\varvec{y}}\right){\mathrm{d}}\boldsymbol{\vartheta }$ and $\pi \left({\vartheta }_{k} |{\varvec{y}}\right)\approx \int \tilde{\pi }\left(\boldsymbol{\vartheta }|{\varvec{y}}\right){{\mathrm{d}}\boldsymbol{\vartheta }}_{-k}$ where ${\boldsymbol{\vartheta }}_{-k}$ is the vector of all hyperparameters $\boldsymbol{\vartheta }$ but with the $k$th hyperparameter ${\vartheta }_{k}$ removed.

## Results

### Prevalence of childhood overweight and obesity in Namibia

Table 1 shows the trends in prevalence rates for childhood overweight and obesity in Namibia from 2000 to 2013. The prevalence rate of childhood overweight (including obesity) increased from 2.0% in 2000 to 5.8% in 2006 and then slightly reduced to 5.3% in 2013. Despite this small drop in 2013, childhood overweight (including obesity) is alarmingly becoming a major overnutrition burden in Namibia because it was above 5% by the year 2013 which was slightly below the worldwide prevalence of 7% in 2012 [1].

**Table 1:** Prevalence trends for childhood overweight and obesity in Namibia from 2000 to 2013

Year	Total	Childhood overweight		Childhood obesity	
		Overweight	Prevalence (%)	Obese	Prevalence (%)
2000	1001	15	0.5	5	0.5
2006	2443	108	1.4	33	1.4
2013	1216	53	0.9	11	0.9

### Selection of latent models and prior distributions

There are several Bayesian modell selection criteria including Akaike information criterion (AIC), Bayesian information criterion (BIC), Bayes factor (BF), Cross-validation (CV), Deviance information criterion (DIC), Log marginal likelihood (LML) computed from Conditional predictive ordinates (CPO), Log pseudo marginal likelihood (LPML) also referred to as the minus log-score, Posterior predictive modell selection (PPMS), and Watanabe–Akaike information criterion (WAIC) [16, 17]. In this study, only four of these (DIC, WAIC, LPML, and LML) were considered because they are available in R-INLA package and are easily computed upon setting the “control.compute” option to “TRUE”.

Tables 2 and 3 present measures of goodness of fit for priors on childhood overweight (including obesity) and estimates for precisions of modell hyperparameters for childhood overweight (including obesity), respectively.

**Table 2:** Measures of goodness of fit for priors on childhood overweight (including obesity)

	Prior	DIC	WAIC	− LPML	− LML
Fixed	Gaussian	36,904.39	36,909.63	18,454.81	18,528.25
	Logit-beta	38,241.08	38,240.73	19,120.36	19,204.35
	Log-gamma	38,318.55	38,318.23	19,159.12	19,243.62
Age of child	RW1	38,096.19	38,117.37	19,058.70	19,232.02
	RW2	13,951.93	29,490.57	16,281.26	18,289.26
Preceding birth interval	RW1	10,041,506	3,211,430	1,181,462	8,199,209
	RW2	21,283,084	2,996,859	1,211,430	11,380,361
Spatial	ICAR	36,096.32	36,150.31	18,075.18	18,743.08
	PCAR	32,757.23	32,764.01	16,382.00	16,402.15
	IID	32,776.37	32,780.45	16,390.28	16,402.56
Temporal	RW1	68,122.75	68,169.99	34,084.97	36,123.68
	RW2	8,428,762	4,878,630	2,432,435	4,215,689

**Table 3:** Estimates for precisions of modell hyperparameters for childhood overweight (including obesity)

	Prior	Posterior mean	Standard deviation	95% credible interval	
Age of child	RW1	22.4931	0.0059	22.4800	22.5068
	RW2	54.5967	0.0018	54.5926	54.5993
Preceding birth interval	RW1	54.5981	0.0001	54.5979	54.5984
	RW2	54.5967	0.0017	54.5929	54.5993
Spatial	ICAR	2.3192	1.4113	0.5772	5.8901
	PCAR	0.2243	0.0003	0.2242	0.2244
	IID	16,562.75	867,028.60	0.0005	32,797.62
Temporal	RW1	2.1889	0.0002	2.1885	2.2325
	RW2	56.3504	0.0012	56.3481	56.3528

We selected the latent models and prior distributions for childhood overweight (including obesity) models as follows. For fixed effects, we selected the Gaussian prior because it had smallest DIC = 36,904.39, smallest WAIC = 36,909.63, smallest − LPML = 18,454.81, and smallest − LML = 18,528.25 (Table 2). For nonlinear effects of age of child, we chose the RW2 prior simply because it had smaller DIC = 13,951.93, smaller WAIC = 29,490.57, smaller − LPML = 16,281.26, and smaller − LML = 18,289.26 (Table 2) and smaller SD = 0.0018 (Table 3). For nonlinear effects of preceding birth, we preferred the RW1 prior as it had smaller DIC = 10,041,506, smaller − LPML = 1,181,462, smaller − LML = 8,199,209, and smaller SD = 0.0001 (Tables 2, 3). For temporal effects, we favoured the RW1 prior because it had smaller DIC = 68,122.75, smaller WAIC = 68,169.99, smaller − LPML = 34,084.97, smaller − LML = 36,123.68, and precision with smaller SD = 0.0002 (Tables 2, 3). For structured spatial effects, the PCAR prior was preferred because it generated a precision with the least standard deviation (SD = 0.0003 (Table 3)) and that it had smallest DIC = 32,757.23, smallest WAIC = 32,764.01, smallest − LPML = 16,382, and smallest − LML = 16,402.15 (Table 2).

### Fixed effects on childhood overweight and obesity in Namibia

Table 4 summarises the estimated fixed effects together with their 95% credible intervals on childhood overweight (including obesity) in Namibia between 2000 and 2013. We observed that urban households, improved water sources, improved toilets, availability of television, male-headed households, and male children were significantly associated with increased childhood overweight (including obesity) in Namibia from 2000 to 2013. Similarly, the household wealth quintile index data were not captured (NA) in the year 2000, and hence, no wealth effects were computed in 2000. We noticed that, in the year 2000, the children whose mothers attained either primary or higher education were significantly associated with reduced childhood overweight (including obesity) whereas those whose mothers attained secondary education were significantly associated with high levels of overweight (including obesity). Finally, we found that childhood overweight (including obesity) significantly increased as the household quintile index, and mother’s education level increased for the years 2006 and 2013.

**Table 4:** Fixed effects on childhood overweight (including obesity) in Namibia from 2000 to 2013

Dummy	2000			2006			2013		
	Posterior mean	95% credible interval		Posterior mean	95% credible interval		Posterior mean	95% credible interval	
(Intercept)	4.26	4.08	4.44	13.30	13.21	13.38	10.76	10.64	10.88
Urban	8.41	8.11	8.71	12.75	12.64	12.86	5.05	4.89	5.21
Water	4.08	3.88	4.27	8.58	8.49	8.66	6.83	6.71	6.96
Toilet	2.57	2.26	2.87	4.67	4.53	4.81	4.80	4.60	5.00
Television	7.28	7.01	7.57	5.05	4.93	5.18	5.65	5.51	5.80
Male head	3.27	3.06	3.48	5.13	5.04	5.22	6.35	6.22	6.48
Male child	2.77	2.66	2.88	7.73	7.64	7.83	6.40	6.27	6.54
Poor	NA	NA	NA	6.76	6.63	6.89	9.76	9.57	9.96
Middle	NA	NA	NA	7.04	6.91	7.17	11.76	11.57	11.94
Richer	NA	NA	NA	6.99	6.84	7.13	9.45	9.25	9.65
Richest	NA	NA	NA	18.02	17.82	18.23	8.61	8.32	8.90
Primary	− 1.59	− 1.87	− 1.32	7.71	7.58	7.83	5.08	4.86	5.29
Secondary	8.09	7.82	8.36	7.58	7.47	7.70	10.98	10.83	11.14
Higher	− 5.44	− 6.46	− 4.47	32.92	32.59	33.26	0.91	0.48	1.34

### Nonlinear effects on childhood overweight and obesity in Namibia

**Fig. 2:**
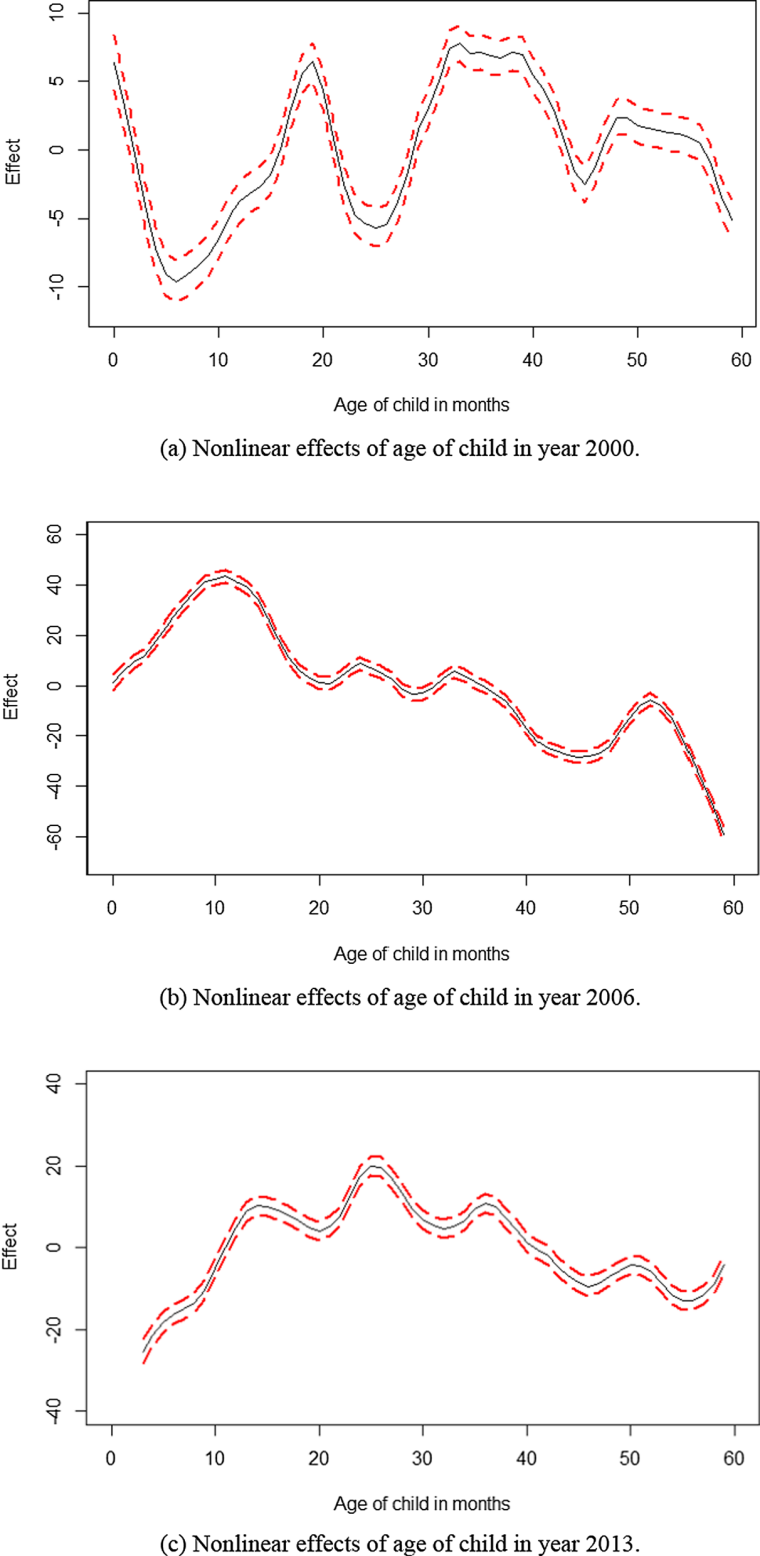
Nonlinear effects of age of child in months on childhood overweight (including obesity) in Namibia from 2000 to 2013

Figure 2 displays the nonlinear effects of age of child on overweight (including obesity) in Namibia from 2000 to 2013. In general, these effects portrayed either an inverse U-shape or an m-shape in such a way that overweight (including obesity) was a major burden within the first 24 months. In the year 2000, overweight (including obesity) depicted a first U-shape during the first 18 months with a major trough centred around 6 months, followed by a second U-shape between 18 and 32 months a medium trough centred around 24 months, then a third U-shape between 36 and 48 months with a minor trough centred around 45 months, and then steadily continued decreasing onwards (Fig. 2a). In the year 2006, overweight (including obesity) exhibited an inverse U-shape during the first 18 months with a crest centred around 10 months and then steadily decreased up to 59 months (Fig. 2b). In the year 2013, overweight (including obesity) also followed a general inverse U-shape with a crest centred around 25 months (Fig. 2c).

**Fig. 3:**
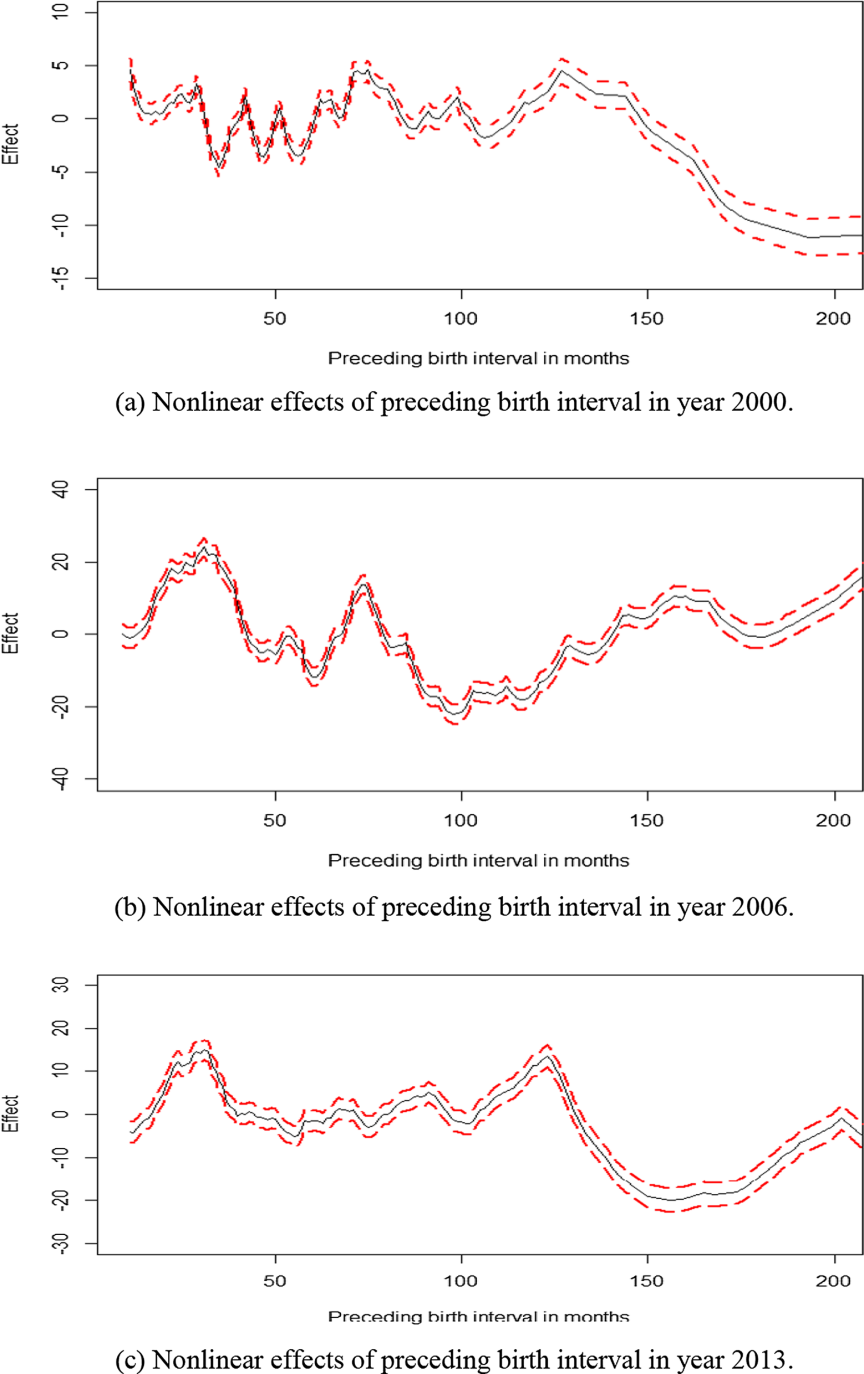
Nonlinear effects of preceding birth interval in months on childhood overweight (including obesity) in Namibia from 2000 to 2013

Figure 3 depicts the nonlinear effects of preceding birth interval on overweight (including obesity) in Namibia from 2000 to 2013. In general, the preceding birth intervals below 36 months (below 3 years) were associated with increased overweight (including obesity) problems and whereas those longer than 60 months (above 5 years) corresponded to reduced levels of overweight and obesity (Fig. 3a, b, c).

### Temporal effects on childhood overweight and obesity in Namibia

**Fig. 4:**
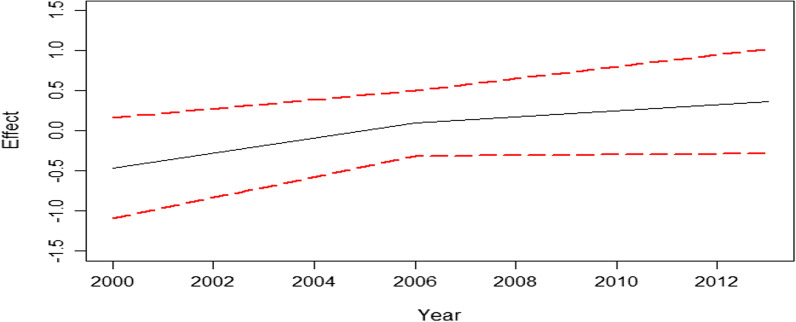
Temporal effects on childhood overweight (including obesity) in Namibia from 2000 to 2013

The temporal effects on overweight (including obesity) in Namibia between 2000 and 2013 are shown in Fig. 4. The effects significantly increased between 2000 and 2006 and then increased further between 2006 and 2013 implying that overweight (including obesity) significantly kept on increasing from 2000 to 2013.

### Spatial effects on childhood overweight and obesity in Namibia

**Fig. 5:**
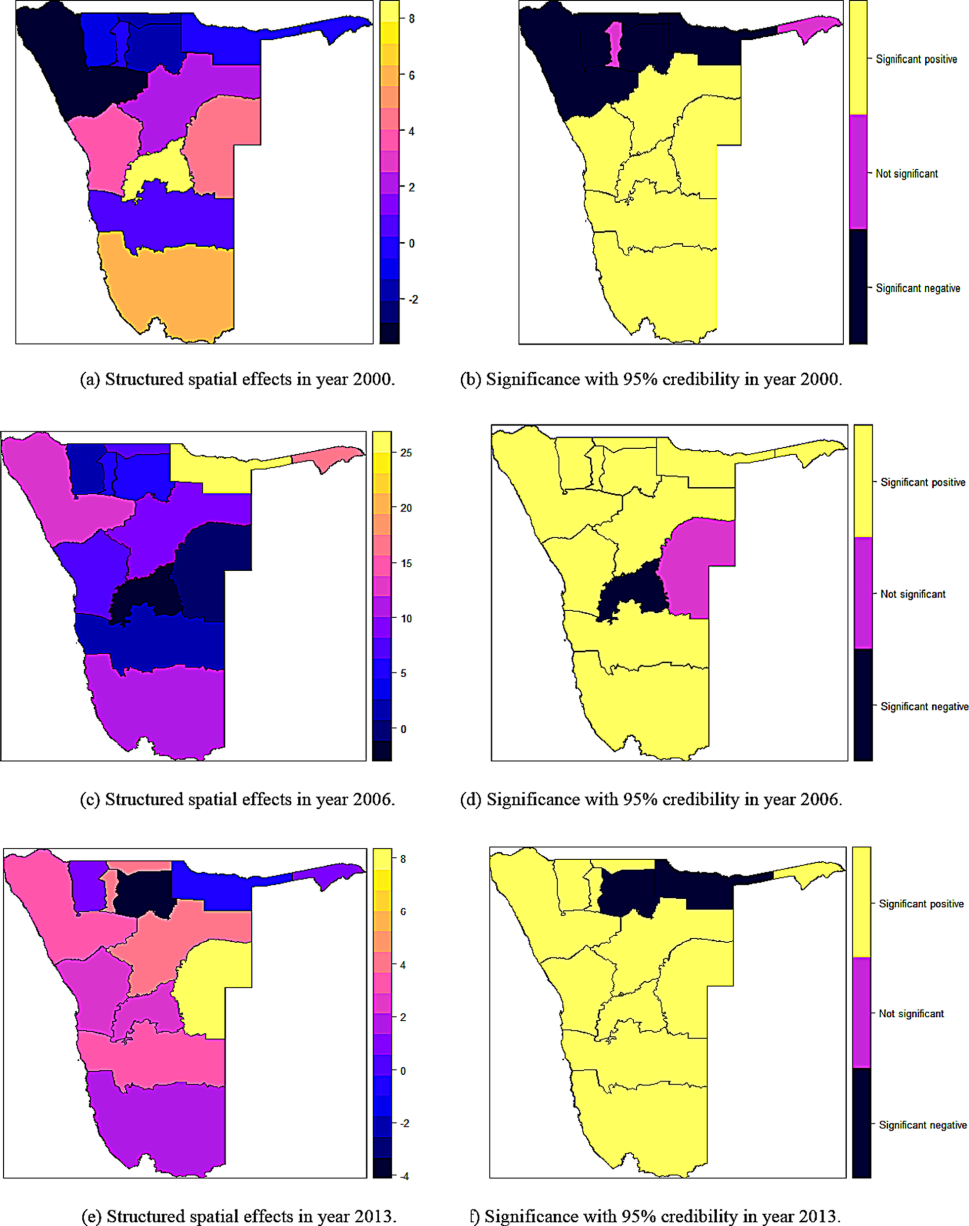
Structured spatial effects and their significance on childhood overweight (including obesity) in Namibia from 2000 to 2013

Figure 5 displays the structured spatial effects on overweight (including obesity) in Namibia between 2000 and 2013. The maps on the left show the posterior means whereas those on the right show the significance of these effects with 95% credibility. A range of colours from black (strongest negative) to yellow (strongest positive) were used to display the intensity of spatial effects within regions. Only three colours were used for discriminating significance of the effects. Firstly, black colour (− 1) corresponded to significant negative structured spatial effects on overweight (including obesity). Secondly, yellow colour (+ 1) corresponded to significant positive structured spatial effects on overweight (including obesity). Lastly, purple colour (0) corresponded to non-significant structured spatial effects on overweight (including obesity). The significant positive structured spatial effects were observed in 6 regions of Namibia; Erongo, Khomas, Otjozondjupa, Omaheke, Hardap, and Karas in the year 2000, in 11 regions of Namibia; Zambezi, Kavango, Ohangwena, Oshana, Omusati, Kunene, Oshikoto, Erongo, Otjozondjupa, Hardap, and Karas in the year 2006, and in 11 regions of Namibia; Zambezi, Ohangwena, Oshana, Omusati, Kunene, Erongo, Omaheke, Khomas, Otjozondjupa, Hardap, and Karas in the year 2013 (Fig. 5b, d, f). Furthermore, it was found that 2 regions; Khomas and Karas were severely associated with increased overweight (including obesity) levels in the year 2000 (Fig. 5a), only 1 region; Kavango was severely associated with increased overweight (including obesity) levels in the year 2006 (Fig. 5c), and only 1 region; Omaheke was severely associated with increased overweight (including obesity) levels in the year 2013 (Fig. 5e). The significant negative structured spatial effects on overweight (including obesity) were observed in 5 regions of Namibia; Kavango, Oshikoto, Ohangwena, Kunene, and Omusati in the year 2000, in only 1 region of Namibia; Khomas in the year 2006, and in 2 regions of Namibia; Kavango and Oshikoto in the year 2013 (Fig. 5b, d, f). It was also found out that 2 regions; Kunene and Omusati were associated with least overweight (including obesity) levels in the year 2000 (Fig. 5a), only 1 region; Khomas was associated with least overweight (including obesity) levels in the year 2006 (Fig. 5c), and only 1 region; Oshikoto was associated with least overweight (including obesity) levels in the year 2013 (Fig. 5e). In the year 2000, Oshana and Zambezi had negative but not significant structured spatial effects on overweight and obesity (Fig. 5b). In the year 2006, Omaheke also had negative but not significant structured spatial effects on overweight and obesity (Fig. 5d).

## Discussion

The inference used in this study was fully Bayesian, and the posterior marginal distributions were estimated using R-INLA package in R 3.5.1. The INLA approach was chosen because it is generally faster than MCMC approach for quantile models. The spatio-temporal quantile interval regression models were fitted for childhood stunting and overweight (including obesity) using the quantile interval approach with the prior distributions preselected by appropriate Bayesian modell selection criteria.

Most of our findings in this study were similar to published findings in related studies within SSA. For example, we previously analysed the 85th quantile response (i.e. at $\tau =0.85$) of childhood overweight in Malawi using 2010 DHS dataset [18]. Just like in our current study, the R-INLA package was used to fit quantile regression models, and it was also observed that urban households, improved drinking water, improved toilet facility, availability of TV, households with male heads, children with male sex, households with higher wealth indexes, and mothers with higher education were associated with significantly increased overweight (excluding obesity) in Malawi.

However, there are minor differences between the two studies, in terms of statistical approaches, as follows. Firstly, in addition to effects of child's age, our current study also considered the effects of PBI on overweight (including obesity). Secondly, our current study analysed quantile interval outcomes of overweight (including obesity) in the interval $0.85\le \tau \le 0.99$ whereas our former study only considered 85th quantile models. Intuitively, our current method is more relevant for modelling overweight and obesity than the former one because it is more efficient than any other quantile-specific models in the interval $0.85\le \tau \le 0.99$.

Note that our current approach is more appropriate for modelling childhood overweight and obesity than the former one because it estimates the pooled modell effects (fixed effects, nonlinear effects, spatial effects, and temporal effects) based on all quantile-specific models within a desired response quantile interval rather than merely basing on a single response quantile level.

Naturally, it appeared awkward and controversial that improved drinking water, improved toilet facility, and higher mother’s formal education were found to be significantly associated with increased childhood overweight in Namibia and in Malawi [18]. However, these findings were quite in order, and their validity can be supported as follows. Higher mother’s formal education was found to be significantly associated with increased childhood overweight and obesity in Hai Phong city in Vietnam because highly educated mothers had highly paid jobs such that they had very little time to spend with their own children and provide them with healthier foods and instead had to rely on outdoor fast foods [19]. Similarly, majority of the children whose mothers were highly educated in Namibia were deprived of healthier foods and appropriate health family lifestyles as they lived with household maids most of the times. Although we did not come across any literature to support our contradicting results on improved drinking water and toilet facility, the main reason could be that these factors were confounded by some other factors like type of residence and household wealth as the children with improved drinking water and toilet facility more likely resided in urban settlements and belonged to rich households.

Furthermore, we ever used quantile interval estimation technique in modelling childhood malnutrition in Republic of Congo [20] but without considering assessment of latent models and prior distributions. Instead of simply choosing latent models and prior distributions at random, we deployed rigorous sensitivity analysis on choice of most appropriate latent models and prior distributions in our current study.

One of the obvious limitations in our study is that all the associations reported in this paper were merely statistical such that no further biological or epidemiological theories were accounted for. Therefore, there is a need for further research to determine whether the significant determinants identified in this study are causal factors or confounding factors of childhood malnutrition in Namibia. If they are merely proximate determinants, then more research must be done to identify the actual causes of childhood stunting and overweight (including obesity) in Namibia. Another important area for further research is identification of possible underlying causes for an alarming increase in prevalence rates of childhood overweight and obesity parallel to the usual undernutrition burden in Sub-Saharan Africa.

## Conclusions

The prevalence rates of childhood overweight (including obesity) in Namibia increased from 2.0% in 2000 to 5.8% in 2006 and then slightly dropped to 5.3% in 2013. The rural residence, improved drinking water, improved toilet facility, availability of television, male-headed households, male children, higher household wealth indexes, and higher mother’s formal education levels were significantly associated with increased childhood overweight (including obesity) in Namibia between 2000 and 2013. It was also noticed that childhood overweight (including obesity) increased as the age of child increased in the first 24 months (in the first 2 years) and decreased from 24 months onwards whereas childhood overweight (including obesity) decreased as the preceding birth interval increased over the years between 2000 and 2013. Furthermore, almost all the regions of Namibia were significantly associated with high levels of childhood overweight (including obesity) in all the 3 years 2000, 2006, and 2013 except for 7 regions; Zambezi, Oshana, Kavango, Oshikoto, Ohangwena, Kunene, and Omusati in 2000, 2 regions; Khomas and Omaheke in 2006, and 2 regions; Oshikoto and Kavango in 2013.

We recommend that scaling-up nutrition programmes and childhood malnutrition policy makers should consider timely interventions based on important socio-demographic factors, child and maternal factors, temporal and spatio-temporal variations of childhood overweight (including obesity) in Namibia as reported in this paper.

## Data Availability

The datasets analysed during the current study are available in the “Measure DHS Program” repository, https://dhsprogram.com/data/available-datasets.cfm. Note that DHS datasets are publicly available on this website but that are downloadable only upon request to the Measure DHS.

## Abbreviations

AIC: Akaike information criterion
BIC: Bayesian information criterion
BF: Bayes factor
BMIAZ: Body mass index-for-age *z*-score
CPO: Conditional predictive ordinate
CV: Cross-validation
DHS: Demographic and Health Survey
GMRF: Gaussian Markov random field
DIC: Deviance information criterion
ICAR: Intrinsic conditional autoregressive
INLA: Integrated nested Laplace approximations
LML: Log marginal likelihood
LPML: Log pseudo marginal likelihood
MCMC: Markov chain Monte Carlo
NCD: Non-communicable diseases
PCAR: Proper conditional autoregressive
PPMS: Posterior predictive modell selection
RW2: Random walk order 2
SD: Standard deviation
SUN: Scaling up nutrition
SUR: Seemingly unrelated regression
SSA: Sub-Saharan Africa
UNICEF: United Nations Children’s Fund (formerly United Nations International Children’s Emergency Fund)
WAIC: Watanabe–Akaike information criterion
WFP: World Food Programme
WHO: World Health Organisation
